# Influence of medial epicondyle morphology on the contribution of flexor–pronator muscle contractions to dynamic elbow stability

**DOI:** 10.1016/j.xrrt.2026.100680

**Published:** 2026-02-09

**Authors:** Koki Orimoto, Emi Nakamura, Yuki Someya, Yuki Shiota, Kohei Kishimoto, Masashi Aoyagi, Takumi Inoue, Yuji Takazawa

**Affiliations:** a Graduate School of Health and Sports Science, Juntendo University, Chiba, Japan; b Department of Rehabilitation, Funabashi Orthopaedic Hospital, Funabashi, Chiba, Japan; c Department of Physical Therapy, Faculty of Health Science, Juntendo University, Tokyo, Japan; d Department of Sports Medicine, Juntendo University, Tokyo, Japan; e Japan Institute of Sports Sciences, Tokyo, Japan; f Department of Sports Medicine and Sportology, Graduate School of Medicine, Juntendo University, Tokyo, Japan; g Institute of Health and Sports Science and Medicine, Juntendo University, Chiba, Japan

**Keywords:** Medial epicondyle, Dynamic elbow stability, Flexor–pronator muscles, Ultrasonography, Baseball players, Moving Valgus Stress Test

## Abstract

**Background:**

Elbow injuries are common among baseball players, often involving the medial epicondyle of the humerus in childhood and the ulnar collateral ligament in adulthood. During skeletal immaturity, excessive valgus stress may lead to fragmentation or hypertrophy of the medial epicondyle. If such morphological abnormalities persist into adulthood, they may compromise elbow stability. The forearm flexor–pronator muscles contribute to dynamic elbow stability under valgus stress during throwing. However, the influence of medial epicondyle morphology on this stabilization remains unclear. This study aimed to clarify the influence of medial epicondyle morphological variations on the dynamic stability of the elbow joint, range of motion, and clinical signs of valgus instability by comparing participants classified according to ultrasonography.

**Methods:**

The medial epicondyle morphology in 48 male collegiate baseball players (19.3 ± 0.9 years; years of athletic experience, 11.6 ± 0.6 years) was classified into 3 groups based on ultrasonographic evaluation: normal, hypertrophic, and fragmented. Dynamic elbow stability was evaluated by measuring the medial joint space distances in both horizontal and vertical directions using ultrasonography, under 3 conditions: rest (gravity eliminated), gravity stress, and contraction of the flexor–pronator muscles. Elbow range of motion and clinical signs of valgus instability were evaluated using the Moving Valgus Stress Test and Elbow Valgus Stress Test. Joint space distances were analyzed using a two-way mixed-design analysis of variance, with morphology and condition as factors. Group differences in range of motion and test outcomes were assessed using one-way analysis of variance or chi-squared tests, as appropriate.

**Results:**

Among the 48 players, 29 were classified as normal (60.4%), 7 as hypertrophic (14.6%), and 12 as fragmented (25.0%). The fragmented group exhibited significantly smaller vertical joint space distances during muscle contraction compared with the normal group, indicating a reduced ability of the forearm muscles to resist valgus stress by medially displacing the ulna. This group also showed significantly limited elbow extension and a higher positive rate on the Moving Valgus Stress Test.

**Conclusion:**

Fragmentation of the medial epicondyle may contribute to compromised medial elbow dynamic stability in adult baseball players. These findings highlight the potential long-term consequences of unresolved fragmentation and support the importance of early detection.

Medial elbow instability is common in baseball players due to repetitive valgus loading during the throwing motion. However, the mechanism leading to medial elbow instability is poorly understood. The medial humeral epicondyle and ulnar collateral ligament (UCL) of the elbow joint are particularly susceptible to valgus stress during throwing motions. The UCL is the primary static stabilizer of the medial elbow and originates from the medial epicondyle.^6^ The medial epicondyle fuses at the age of approximately 15–16 years. In school-aged children, skeletal immaturity increases the risk of medial epicondylar apophysitis and avulsion fractures.^7^^,^^22^ They may be accompanied by elbow pain, limited range of motion (ROM), and secondary issues, such as osteophyte formation and posteromedial impingement during healing.^13^^,^^15^

Muscle contractions of the forearm flexor–pronator muscles (FPMs), particularly the flexor digitorum superficialis and flexor carpi ulnaris, contribute to the stability of the elbow joint.^12^ The tendinous complex, consisting of the FPMs and its deep aponeuroses, attaches near the medial epicondyle and integrates with the joint capsule.^12^ This attachment site with tendon, ligament, or joint capsule inserts into the bone and acts to transmit tensile load from soft tissues to the bone.^2^ This structure supports the UCL and acts as a dynamic stabilizer to resist valgus stress during throwing motions. Previous studies have evaluated the dynamic stability of the elbow joint provided by the FPMs using ultrasonography by measuring the medial joint space during muscle contraction.^11^^,^^23^^,^^29^ Although previous studies have examined the dynamic stability of the FPMs, these studies have generally focused on muscle activity or joint space distance change without considering bony morphology. Despite the anatomical significance of the medial epicondyle as the origin of key stabilizing muscles, little attention has been paid to the effect of its morphological variations on dynamic stability.

Excessive valgus stress is the primary mechanical factor causing medial elbow injuries, particularly in collegiate and professional baseball players.^8^^,^^16^ Many professional baseball players have reported that they started specialized training by the age of 8–9 years and often play for over a decade.^31^ If morphological abnormalities of the medial epicondyle persist into adolescence or adulthood, they may compromise the dynamic stability provided by the FPMs at their attachment site, leading to increased mechanical loading of the UCL and progressively contributing to the development of medial elbow instability. We hypothesized that variations in the morphology of the medial humeral epicondyle may affect the dynamic stability of the elbow joint. This study aimed to clarify the influence of medial epicondyle morphology on the dynamic stability of the elbow joint, ROM, and clinical signs of valgus instability by comparing groups classified by ultrasonography.

## Materials and methods

### Study design

This observational cross-sectional study was conducted in a controlled university laboratory setting (temperature and lighting) in July 2024, during a baseball season.

### Participants

**Figure 1:**
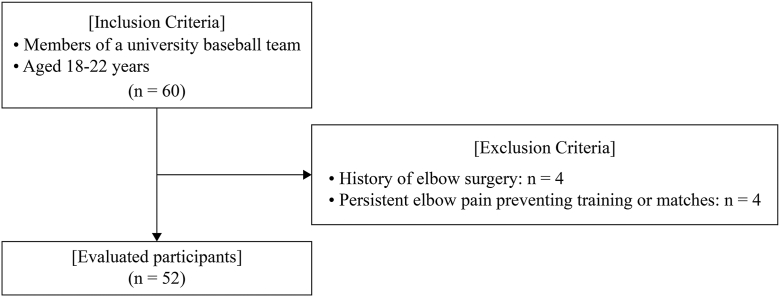
Flowchart of participant selection. Sixty male collegiate baseball players were initially recruited. Eight participants were excluded based on the exclusion criteria: 4 had a history of elbow surgery, and 4 had persistent elbow pain preventing participation in training or matches. Fifty-two participants were evaluated.

This study initially recruited 60 male collegiate baseball players from a single university baseball team. Adult baseball players who started playing baseball before the age of 15 years, the approximate age at which the medial epicondyle typically fuses, were included in this study.^7^ The exclusion criteria included a history of elbow surgery and current elbow pain preventing participation in training or matches. Among the 60 players initially recruited, 8 were excluded based on the exclusion criteria. Specifically, 4 players had a history of elbow surgery, and 4 players reported persistent elbow pain that prevented participation in training or matches. After applying the exclusion criteria, 52 players remained eligible and were included in this study. A flowchart detailing the participant recruitment and selection process is provided in Figure 1.

### Measurements

All participants received ultrasonographic evaluation and physical examination. Prior to this examination, demographic information, including age, height, weight, medical history, and years of sports participation and injury information evaluated by the Japanese version of the Oslo Sports Trauma Research Center Questionnaire,^19^ was obtained through an online Google Form. The participants maintained their usual routines with no activity restrictions before the examinations.

### Ultrasonographic evaluation

A single trained physical therapist performed all ultrasonographic evaluations using a SONIMAGE MX1 (KONICA MINOLTA, Tokyo, Japan) scanner with an 11-MHz linear transducer.

### Medial epicondyle morphology

**Figure 2:**
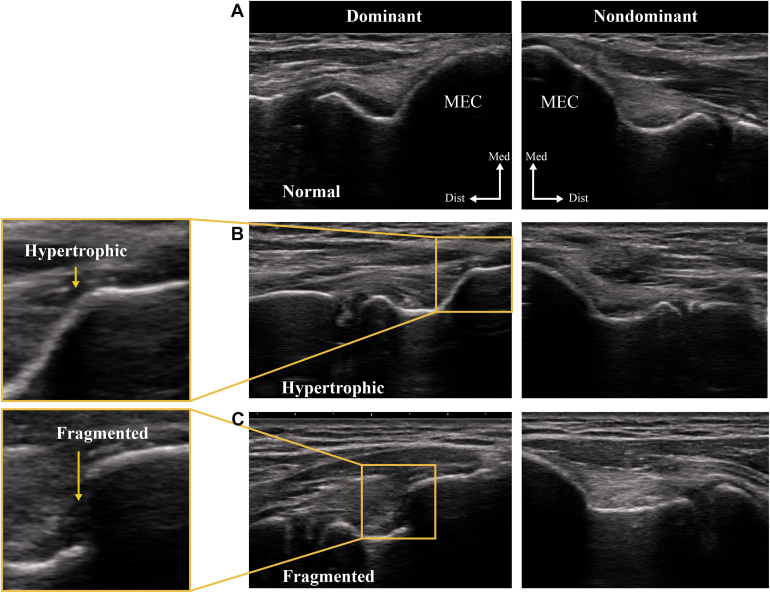
Classification of medial humeral epicondyle morphology. The MEC was evaluated using ultrasonography in the long-axis view. Morphology was classified into 3 categories: (**A**) normal group, showing an appearance identical to that of the nonthrowing side; (**B**) fragmented group, showing a clear discontinuity of the cortical margin; and (**C**) hypertrophic group, showing a distinct cortical protrusion.^30^*MEC*, medial epicondyle; *Dist*, distal; *Med*, medial.

The medial epicondyle of the humerus was evaluated in the long-axis view. Morphology and continuity of the cortical margin were assessed. The medial epicondyle morphology was classified into 3 groups: normal, fragmented, and hypertrophic. The normal group showed an appearance identical to that of the nonthrowing side; the fragmented group exhibited a clear discontinuity of the cortical margin; and the hypertrophic group showed a distinct cortical protrusion. This classification was based on the criteria described by Watanabe et al^30^ and is illustrated in Fig. 2.

### Measurement of joint space distance

**Figure 3:**
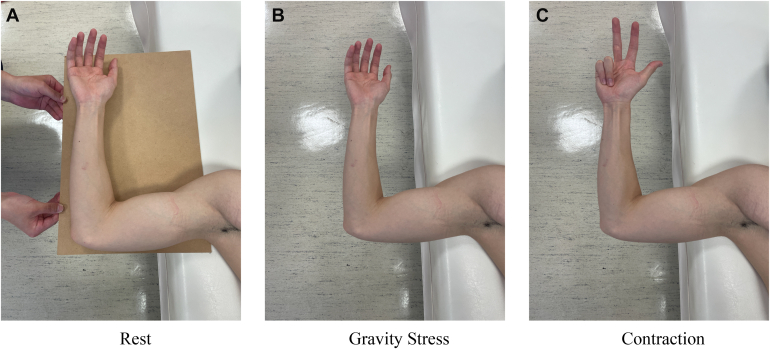
Measurement of medial joint space distance of the elbow under different conditions. Ultrasonographic evaluation of the medial joint space distance under 3 conditions: (**A**) at rest (gravity eliminated), (**B**) with gravity stress, and (**C**) during contraction of the forearm flexor–pronator muscles (performed by flexing the fourth and fifth proximal interphalangeal joints).

**Figure 4:**
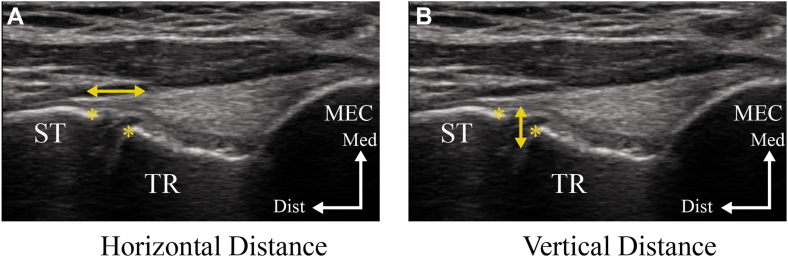
Measurement of medial joint space distance using ultrasonography. The *yellow lines* indicate the 2 anatomical landmarks: the distal–medial corner of the humeral trochlea and the proximal edge of the sublime tubercle of the ulna (*asterisks*). (**A**) Horizontal distance: measured parallel to the joint line. (**B**) Vertical distance: measured perpendicular to the joint line. *MEC*, medial epicondyle; *ST*, sublime tubercle; *TR*, trochlea; *Dist*, distal; *Med*, medial.

The dynamic stability of the medial elbow was assessed with the participant in the supine position, shoulder abducted to 90°, elbow flexed to 90°, and the forearm in a neutral position. This elbow flexion angle was selected because a previous study demonstrated that the medial joint space was significantly larger at 90° than at 30°, suggesting 90° is suitable for identifying valgus instability.^14^ Imaging was performed under 3 conditions: rest (gravity eliminated), gravity stress, and muscle contraction (flexion of the fourth and fifth proximal interphalangeal joints) (Fig. 3).^25^ To evaluate medial joint stability, we measured both horizontal and vertical joint space distances from still ultrasound images using the ImageJ software, and the absolute values under each condition were used for analysis. This method was adapted from Sasaki et al,^26^ who originally proposed this measurement approach to assess medial elbow laxity in baseball players using ultrasonography. The measurements were based on 2 anatomical landmarks: the distal–medial corner of the humeral trochlea and the proximal edge of the sublime tubercle of the ulna. These landmarks were selected based on their consistent visibility on ultrasound and their relevance to the UCL. The horizontal distance (measured parallel to the joint line) was defined as the linear distance between the anatomical landmarks. An increase in horizontal distance was interpreted as medial joint space widening. The vertical distance was defined as the perpendicular distance from the humeral trochlea to the sublime tubercle, reflecting ulnar displacement relative to the humerus. A decrease in vertical distance indicated lateral displacement, whereas an increase indicated medial displacement. Under valgus stress induced by gravity, horizontal distance typically increased while vertical distance decreased. Therefore, a reduction in horizontal distance and an increase in vertical distance with muscle contraction were interpreted as dynamic stability of the medial elbow. Figure 4 illustrates the measurement method. The intrarater reliability for measuring joint space distances showed high intraclass correlation coefficients across all conditions, ranging from 0.984 to 0.986 for horizontal distance and 0.985 to 0.990 for vertical distance, with 95% confidence intervals between 0.974 and 0.994.

### Physical examination

ROM, the Elbow Valgus Stress Test, and the Moving Valgus Stress Test (MVST) were each performed once per participant by 1 of 2 trained physical therapists. Each participant was seated in a standardized upright seated position. Elbow flexion, extension, forearm pronation, supination, and wrist flexion and extension were measured bilaterally using a goniometer, and the maximum active range of each motion was recorded. To evaluate valgus instability of the elbow, the Elbow Valgus Stress Test was performed with the participant's elbow flexed to approximately 20° while the examiner palpated the medial joint line. One hand stabilized the distal humerus, and the other applied a valgus force to the elbow. The test was considered positive if medial elbow pain was provoked.^9^ In the MVST, the shoulder was abducted to 90°, and the examiner stabilized the elbow and grasped the distal forearm. With a valgus force applied, the examiner externally rotated the shoulder while maintaining maximal elbow flexion and then rapidly extended to approximately 30°. The test was considered positive if the participant reported medial elbow pain between 70° and 120° of elbow flexion.^21^

### Statistical analyses

Normality of the data was assessed using the Shapiro–Wilk test. The difference in demographic data, ROM, and Oslo Sports Trauma Research Center Questionnaire score among the 3 groups was assessed using one-way analysis of variance (ANOVA) or the Kruskal–Wallis test, as appropriate.

A two-way mixed-design ANOVA was conducted to examine the effects of condition (rest, gravity, and muscle contraction; within-subject factor) and medial epicondyle morphology (normal, hypertrophic, and fragmented; between-subject factor) on medial elbow joint space distances. Post hoc comparisons were performed using Bonferroni correction. The Elbow Valgus Stress Test and MVST results were classified into positive and negative groups, and the 3 groups (normal, hypertrophic, and fragmented) were compared using the chi-squared test, followed by residual analysis. All statistical analyses were performed using IBM SPSS Statistics (Version 29.0; IBM, Armonk, NY), with a significance level set at *P* < .05 for all analyses.

### Ethical consideration

This study was conducted in accordance with the ethical principles outlined in the Declaration of Helsinki and was approved by the appropriate ethics committee. The ethics review process ensured that this study adhered to appropriate guidelines for protecting the rights and welfare of participants. Participation was entirely voluntary, and the participants were explicitly informed that their decision to participate or not would have no impact on their team activities, academic standing, or any other benefits. The study procedures, purpose, potential risks, and benefits were explained directly to each player, and written informed consent was obtained, including handwritten signatures. All collected data were anonymized and securely stored to ensure confidentiality.

## Results

Fifty-two participants initially underwent ultrasonographic measurements; however, 4 participants were subsequently excluded from the final analysis owing to inaccurate measurements. Thus, 48 participants (19.3 ± 0.9 years; height, 173.0 ± 5.6 cm; weight, 72.5 ± 9.9 kg; body mass index, 24.2 ± 2.7; years of athletic experience, 11.6 ± 0.6 years) were evaluated. Using an ultrasound imaging system, the participants were classified into 3 groups based on the morphology of the medial humeral epicondyle: 29 (60.4%) participants in the normal group, 7 (14.6%) in the hypertrophic group, and 12 (25.0%) in the fragmented group. Participants' characteristics and background variables for each morphology group are shown in Table I. No significant difference was observed among the groups (normal, hypertrophic, and fragmented) in demographic and injury information.

**Table I:** Comparison of participant characteristics across groups based on morphology classification∗.

Variable	Normal (n = 29)	Hypertrophic (n = 7)	Fragmented (n = 12)	*P* value
Age, yr	19.1 ± 1.0	19.9 ± 0.7	19.3 ± 0.6	.061
Height, cm	173.3 ± 6.2	171.2 ± 3.6	172.7 ± 5.2	.632
Weight, kg	71.3 ± 9.1	70.4 ± 14.4	74.5 ± 10.2	.600
BMI, kg/m^2^	23.7 ± 2.4	24.0 ± 4.2	25.0 ± 3.0	.423
Experience, yr	11.6 ± 0.3	11.7 ± 0.7	11.6 ± 0.8	.160
Position, n				.714
Pitcher	7 (24.1%)	2 (25.0%)	3 (27.3%)	
Catcher	3 (10.3%)	0 (0%)	2 (18.2%)	
Fielder	19 (65.6%)	6 (75.0%)	6 (54.5%)	
OSTRC, point	3.1 ± 8.0	0.0 ± 0.0	6.7 ± 10.6	.163

*BMI,* body mass index; *OSTRC,* Oslo Sports Trauma Research Center Questionnaire.

∗ The data are expressed as means ± standard deviations.

Comparing the medial elbow joint space distances among the groups, the vertical distance in the muscle contraction condition was significantly smaller in the fragmented group (0.16 ± 0.58 mm) than in the normal group (1.01 ± 0.92 mm) (*P* = .016). The two-way mixed-design ANOVA revealed a significant main effect of condition on horizontal distance (*P* < .001) and significant main effects of both condition (*P* = .002) and morphology (*P* = .029) on vertical distance. However, no significant interaction effects were observed between condition and morphology for either horizontal or vertical distance (Table II).

**Table II:** Comparison of medial elbow joint space distance based on morphology classification†.

Variable	Normal (n = 29)	Hypertrophic (n = 7)	Fragmented (n = 12)	*P* value		
				Morphology	Condition	Interaction
Horizontal distance				.187	<.001	.400
Rest	4.29 ± 0.86	4.23 ± 1.30	4.85 ± 0.97			
Gravity	4.65 ± 1.16	4.45 ± 1.23	5.18 ± 1.03			
Contraction	4.12 ± 1.14	4.06 ± 1.13	4.94 ± 1.07			
Vertical distance				.029	.002	.080
Rest	0.88 ± 0.89	0.63 ± 1.01	0.32 ± 0.63			
Gravity	0.82 ± 0.97	0.21 ± 1.20	0.084 ± 0.70			
Contraction	1.01 ± 0.92	0.64 ± 0.69	0.16 ± 0.58∗			

Pairwise comparisons.

∗ Post hoc Bonferroni test: normal vs. fragmented, *P* = .016.

† The data are expressed as means ± standard deviations.

A significant difference in elbow extension was observed between the morphology groups (*P* = .035). Post hoc analysis using the Bonferroni correction revealed that the fragmented group exhibited significantly lower extension (−5.7 ± 2.7°) compared with the normal group (1.9 ± 1.0°) (*P* = .012). Furthermore, the fragmented group demonstrated a significantly higher positive rate in the MVST than that in the other groups (*P* = .013) (Table IIII).

**Table III:** Comparison of range of motions and clinical signs of valgus instability based on morphology classification‡.

Variable	Normal (n = 29)	Hypertrophic (n = 7)	Fragmented (n = 12)	*P* value
Range of motion				
Elbow flexion	131.6 ± 4.9	140.2 ± 2.5	140.6 ± 1.0	.174
Elbow extension	1.9 ± 1.0	0.3 ± 0.4	−5.7 ± 2.7∗	**.035**
Supination	88.5 ± 9.2	91.9 ± 7.9	90.8 ± 11.6	.608
Pronation	74.3 ± 8.2	75.6 ± 10.5	78.1 ± 7.4	.437
Palmar flexion	66.0 ± 8.8	61.4 ± 7.8	65.8 ± 4.4	.332
Palmar extension	70.8 ± 8.6	68.6 ± 7.2	68.2 ± 10.2	.332
Valgus instability				
Elbow Valgus Stress test	2 (6.8%)	0 (0%)	3 (25.0%)	.180
Moving Valgus Stress test	1 (3.4%)	0 (0%)	4 (33.3%)†	**.013**

Pairwise comparisons.

Boldface *P* values indicate statistically significant difference between the 3 conditions (*P* < .05, one-way analysis of variance or chi-squared test).

∗ Post hoc Bonferroni test: normal vs. fragmented, *P* = .012.

† Residual analysis: fragmented vs. others, *P* = .016.

‡ The data are expressed as means ± standard deviations.

## Discussion

This study investigated the morphological variations of the medial humeral epicondyle and the relationship between the morphology and elbow dynamic stability in adult baseball players. The results showed that 25% of all players had fragmented medial epicondyles, and these players exhibited smaller vertical joint space distances during contraction of the FPMs, higher positive rates in the MVST, and more limited elbow extension ROM than those of other morphological groups. These findings suggest that the morphology of the medial epicondyle can be related not only to the elbow dynamic instability but also to ROM limitation.

Most previous studies investigating the medial epicondyle have primarily focused on school-age baseball players, and no study has addressed its classification or clinical relevance in adult athletes. In younger players, the medial epicondyle epiphysis consists of growth cartilage that has not yet ossified. Inadequate bone development, combined with valgus stress on the elbow and traction from the FPMs, is thought to cause separation and fragmentation of the medial epicondyle.^4^^,^^27^ If repetitive stress persists, medial epicondyle fragmentation may result in nonunion.^3^ The main treatment involves 4–6 weeks of rest and learning proper throwing mechanics.^18^ However, players with mild symptoms may underestimate the need for prolonged rest and return to throwing prematurely. Consequently, the separated epiphyseal cartilage from childhood may remain unfused into adulthood. Harada et al reported that 21% (33 out of 153) of baseball players aged 9–12 years had medial epicondyle fragmentation.^10^ In comparison, the present study found a comparable prevalence of medial epicondyle fragmentation of 25% (12 out of 48) in adult baseball players aged 18–22 years, suggesting that the medial epicondyle fragmentation observed in childhood may persist into adulthood.

Compared with the normal group, the fragmented group exhibited significantly smaller vertical joint space distance during muscle contraction. Players with UCL injuries also show reduced vertical distances during muscle contraction. This reduction reflects impaired medial displacement of the ulna by the FPMs.^20^ The present study showed similar findings in the fragmented group and suggested that medial epicondyle fragmentation may impair medial elbow dynamic stability, as observed in UCL-injured groups. This impaired displacement of the ulna may be due to disrupted force transmission from the FPMs to the medial elbow. The flexor digitorum superficialis is particularly important due to its thick muscle belly and direct attachment to the medial epicondyle, making it a key contributor to elbow joint stability.^24^ Thus, partial detachment of the tendon origin in the fragmented group can impair this transmission and lead to diminished medial displacement of the ulna during contraction. In contrast, horizontal joint distance did not differ among the groups. This finding suggests that valgus stability may be more sensitively reflected by vertical displacement of the ulna during muscle contraction than by horizontal joint widening. Therefore, vertical and horizontal joint space distances should be separately evaluated when assessing medial elbow stability.

Previous studies on baseball players aged 12–18 years have reported no significant differences in elbow ROM between the fragmented and normal groups.^17^ In contrast, the present study found that the fragmented group, consisting of players aged 18–22 years, showed a clear limitation in elbow extension. Furthermore, the fragmented group demonstrated higher positive rates in the MVST. This test is designed to detect valgus instability specifically between 70° and 120° of elbow flexion, which corresponds to the phase of the throwing motion when peak valgus torque typically occurs.^28^ These findings imply that continued pitching without adequate dynamic stability may lead to secondary adaptations, such as limited elbow extension.

The most important finding of this study is that medial epicondyle fragmentation observed in adulthood may have originated from incomplete healing during growth and appears to be associated with reduced medial elbow dynamic stability during muscle contraction. To prevent these issues, early detection and proper care during the growth period are important. In addition, evaluating the morphology of the medial epicondyle remains important even in adulthood to understand potential risks to joint stability.

This study has some limitations. First, whether the observed reduction in dynamic stability occurred before or after the morphological changes remains unclear. This is because the cross-sectional design limits the ability to determine whether a causal relationship exists between medial epicondyle morphology and elbow stability. However, medial epicondyle deformities typically develop during skeletal immaturity before epiphyseal fusion and rarely occur afterward.^1^ Based on this evidence, we believe that the deformities observed in this study likely occurred prior to bony fusion. Second, this study did not assess pitching experience in detail. Pitchers have a higher incidence of medial elbow injuries owing to greater exposure to valgus stress during repeated throwing.^5^ However, the proportion of pitchers did not differ among the morphology groups, indicating that pitching exposure was unlikely to confound the present results. Consequently, the current findings likely reflect a direct relationship between epicondyle morphology and functional joint stability.

## Conclusion

This study identified medial epicondyle fragmentation in 25% of collegiate baseball players. Compared with the normal group, the fragmented group showed reduced vertical distances in the medial elbow joint space during muscle contraction, indicating a decreased ability to medially shift the proximal ulna. Additionally, the fragmented group exhibited significantly lower elbow extension ROM and a higher positive rate in the MVST. These findings highlight the potential long-term consequences of unresolved fragmentation and support the importance of early detection.

## Acknowledgments

The authors sincerely thank Professor Naotoshi Mitsukawa of Toyo Gakuen University for his invaluable support in coordinating the data collection.

## Disclaimers:

Funding: This work was supported by the Joint Research Program of Juntendo University, Faculty of Health and Sports Science. The article processing charge (APC) for Open Access was supported by the Institute of Health and Sports Science and Medicine, Juntendo University.

Conflicts of interest: The authors, their immediate families, and any research foundations with which they are affiliated have not received any financial payments or other benefits from any commercial entity related to the subject of this article.
